# Specialist training in Fiji: Why do graduates migrate, and why do they remain? A qualitative study

**DOI:** 10.1186/1478-4491-7-9

**Published:** 2009-02-12

**Authors:** Kimberly M Oman, Robert Moulds, Kim Usher

**Affiliations:** 1 James Cook University School of Medicine and Dentistry, Townsville, Queensland, Australia; 2 Fiji School of Medicine, Suva, Fiji; 3 James Cook University School of Nursing, Midwifery and Nutrition, Townsville, Queensland, Australia

## Abstract

**Background:**

Specialist training was established in the late 1990s at the Fiji School of Medicine. Losses of graduates to overseas migration and to the local private sector prompted us to explore the reasons for these losses from the Fiji public workforce.

**Methods:**

Data were collected on the whereabouts and highest educational attainments of the 66 Fiji doctors who had undertaken specialist training to at least the diploma level between 1997 and 2004. Semistructured interviews focusing on career decisions were carried out with 36 of these doctors, who were purposively sampled to include overseas migrants, temporary overseas trainees, local private practitioners and public sector doctors.

**Results:**

120 doctors undertook specialist training to at least the diploma level between 1997 and 2004; 66 of the graduates were Fiji citizens or permanent residents; 54 originated from other countries in the region. Among Fiji graduates, 42 completed a diploma and 24 had either completed (21) or were enrolled (3) in a master's programme. Thirty-two (48.5%) were working in the public sectors, four (6.0%) were temporarily training overseas, 30.3% had migrated overseas and the remainder were mostly in local private practice. Indo-Fijian ethnicity and non-completion of full specialist training were associated with lower retention in the public sectors, while gender had little impact. Decisions to leave the public sectors were complex, with concerns about political instability and family welfare predominating for overseas migrants, while working conditions not conducive to family life or frustrations with career progression predominated for local private practitioners. Doctors remaining in the public sectors reported many satisfying aspects to their work despite frustrations, though 40% had seriously considered resigning from the public service and 60% were unhappy with their career progression.

**Conclusion:**

Overall, this study provides some support for the view that local or regional postgraduate training may increase retention of doctors. Attention to career pathways and other sources of frustration, in addition to encouragement to complete training, should increase the likelihood of such programmes' reaching their full potentials.

## Background

Migration of doctors from developing to industrialized countries has accelerated in recent years, and threatens the ability of many underresourced countries to meet the health care needs of their own populations. Shortages of health workers have been identified as major barriers to making progress towards the Millennium Development Goals, and human resource issues are receiving increasing attention at an international level [1,2].

An important approach to increasing the numbers of health workers in developing countries is the "scaling up" of health professional education and training [1,3], including the establishment of in-country and regional specialist training [4]. Postgraduate training has recently been established in Fiji, a small developing Pacific Island nation (see Table 1) [5], in order to address a continuing dependence on expatriates, as well as a failure of most overseas-trained Pacific Island specialists to return home.

**Table 1:** Population [5] and health-related statistics [1] for Fiji origins

Population – 2006	853 000
Indigenous Fijians	55%

Indo-Fijians (Fiji citizens of Indian descent)	37%

Other ethnicities	8%

Gross domestic product (GDP), 2004	USD 3280 per capita

Under-5 mortality, 2004	18 per 1000 live births

Life expectancy at birth, 2004	68 years

Annual health expenditure, 2004	USD 104 per capita

Expenditure as % of GDP, 2004	3.7%

Fiji has a population of 853 000. In recent years the health system has been burdened not only by epidemics of chronic diseases, but by considerable ongoing morbidity and mortality from infectious diseases as well (though there is no malaria transmission, and only 171 HIV cases had been diagnosed between 1989 and 2004) [6]. There are 406 established posts for doctors within the public service, of which 251 (61.8%) were filled by locals, 90 (22.2%) were filled by expatriates and 65 (16.0%) were unfilled in 2006[7].

There is universal access to health care [6], and the vast majority of the population receives inpatient care in the public system. While private general practitioner services have been available for many years, all inpatient specialist services were delivered in public hospitals until the opening of a small private hospital in the capital in 2001.

Postgraduate training was first established at the Fiji School of Medicine (FSMed) in 1998 (1997 for anaesthesia) and consists of a one-year diploma, followed by an additional three years leading to Master's of Medicine (MMed) qualifications in obstetrics and gynaecology, paediatrics, internal medicine, surgery and anaesthesia [8-11]. Although it was believed that offering training in the Pacific and awarding a local specialist qualification not recognized elsewhere would improve retention in the public sectors [12], within a few years, many doctors who had started training were leaving the public system to enter local private practice or to migrate overseas. This was exacerbated around the time of a coup in 2000 which, along with previous coups in 1987, has been particularly associated with migration of Fiji citizens of Indian descent ("Indo-Fijians").

To date, few studies have been published about postgraduate specialist training programmes in developing countries, and these have usually not focused on migration and retention issues [13-19]. A number of surveys and qualitative studies have looked at reasons why doctors migrate or consider migrating out of their countries of origin [20-24], while other studies have explored job dissatisfaction, stress and coping mechanisms [25-35]. Such studies have cited dissatisfaction with finances, living conditions, heavy workloads, poor working conditions, problems with access to training and career progression, dissatisfaction with health management, concerns about family welfare and political instability and security issues, with some variation from country to country.

This study examines the role of a locally-available specialist training programme in both producing new specialists and retaining them in the public practice sector. It also explores some of the factors that have influenced the decisions of doctors, who have completed a local diploma or master's programme, to either remain in or leave public sector practice.

## Methods

One hundred and twenty doctors completed specialist training at least to the first-year diploma level between 1998 (1997 for anaesthesia) and 2004. Of these, 66 were citizens or permanent residents of Fiji, and 54 were from other countries in the region. Quantitative data were collected on the gender, ethnicity, highest educational attainment and working location as of December 2006 for all these doctors. Data were obtained from enrolment and graduation records from the Fiji School of Medicine (FSMed), from local specialist coordinators and from publicly-available medical registration information in New Zealand and Australia, with whereabouts confirmed for all 66 doctors. These data were analysed by means of Epi-Info software [36]. The experiences of 54 trainees from other countries in the region are not presented here.

**Figure 1:**
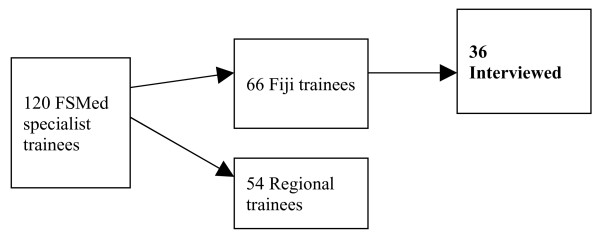
**Flow diagram for the doctors interviewed**.

Qualitative data was obtained from in-person interviews by a single interviewer with 36 of 66 who had undertaken specialist training through FSMed (see Fig. 1). These were carried out during four trips to Fiji and three trips within Australia between April 2004 and September 2006.

The interviews were semistructured and lasted for half an hour to an hour-and-a-half. Doctors were purposively selected for interviewing in order to obtain broad representation on the basis of ethnicity, gender, specialty choice, highest educational attainment and migration status (see Table 2). Although doctors living in Fiji outside of Suva (the capital) as well as doctors living in Australia were interviewed, for practical reasons most doctors were interviewed in Suva (25 out of 36). In particular, migrants, private doctors and doctors who had not completed an MMed were underrepresented due to increased geographical scattering for these groups.

**Table 2:** Characteristics of Fiji School of Medicine specialist trainees 1996–2004 (Fiji graduates only, excluding graduates from other regional countries)

**Characteristic**	**Specialist graduates**		
	
	**All Fiji citizens or permanent residents**	**Number Interviewed**	**% interviewed**
Total	66	36	54.5%

Gender			

Males	39	22	56.4%

Females	27	14	51.9%

Ethnicity			

Fijians	41	23	56.1%

Indo-Fijians	20	10	50.0%

Others	5	3	60.0%

Specialty			

Anaesthesia	11	6	54.5%

Medicine	12	8	66.7%

Obstetrics and Gynaecology	14	6	42.9%

Paediatrics	18	9	50.0%

Surgery	11	7	63.6%

Highest educational attainment as of December 2006			

Diploma	42	14	33.3%

Master's (21) or MMed (3) student	24	22	91.7%

Working status			

Public sectors (Ministry of Health, FSMed, or UN)	32	21	65.6%

Temporarily overseas	4	4	100%

Private (9) or not working (1) – Fiji	10	4	40%

Overseas migrants	20	7	35%

As part of the interviews, doctors working in the public sectors, which is defined as being employed by the Ministry of Health (29), the Fiji School of Medicine (2) or by a United Nations organization (1), and doctors temporarily training overseas but still employed by the Ministry of Health (3 out of 4) were asked about their reasons for remaining in the public sector, as well as whether they had considered resigning. Doctors in private practice as well as overseas migrants were asked to describe their decisions to leave the public sector.

The interviews were audiotaped, professionally transcribed and analysed by means of QSR-N6 software [37]. All interview passages were coded into at least one of several dozen codes that were initially derived from the first round of interviews and later refined. Coded passages were sorted for analysis according to working status (public sector, temporarily training overseas, local private practice or overseas migrant). Analysis was carried out by means of a constant comparative method, with emerging themes being tested and refined through returning repeatedly to the interview transcripts. A case study database was created that allows for tracing of findings and interpretations to original data. Ongoing findings were presented for comment to interview participants and other stakeholders at the annual Fiji Medical Association conferences in 2005, 2006 and 2007.

The principal author, who carried out the interviews, worked at FSMed between 1998 and 2001 and played a major role in establishing postgraduate training in internal medicine. During the analysis and interpretive process, the implications, benefits, limitations and potential for bias arising from this semi-insider status were acknowledged, reflected upon and discussed with supervisors.

Ethics approval was obtained from James Cook University (H1743) and the Fiji National Research Ethics Review Committee (005-2004).

The funding sources played no role in the collection, analysis and interpretation of data, in the writing of the report or in the decision to submit the paper for publication.

## Results

Between 1997 and 2004, 120 students had undertaken training to at least the diploma level at the Fiji School of Medicine (FSMed), including 66 graduates who were citizens or permanent residents of Fiji and 54 regional graduates from other Pacific Islands. Among the 66 Fiji graduates, by December 2006, 24 had either completed a master's degree programme (21) or were still enrolled as master's students (3), and 42 had left training with a diploma as their highest qualification. While some doctors enrolled in the diploma programme but did not complete a diploma, the available records were incomplete and otherwise less reliable than for graduates, and therefore these doctors were not included in the study.

**Figure 2:**
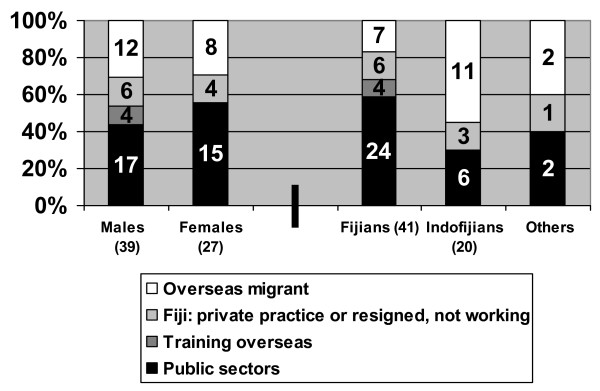
**Working status by gender and ethnicity for specialist graduates of Fiji origins**.

Of the 66 Fiji graduates, 32 (48.5%) were working in the public sectors, and four (6.0%) were training overseas with stated intentions to return as of December 2006. Ten (15.2%) who resigned were still living in Fiji (nine in local private practice and one on temporary maternity leave), while 20 (30.3%) were believed to have permanently migrated overseas (see Fig. 2).

**Figure 3:**
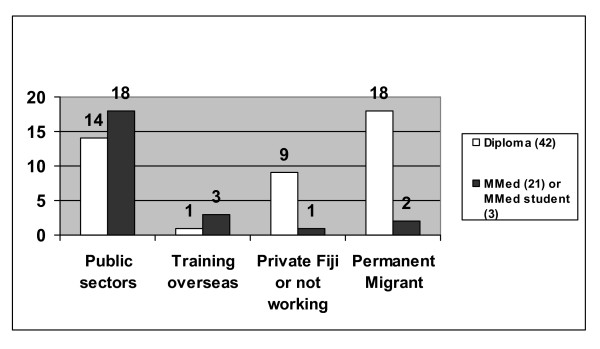
Working status by highest qualification attained (as of December 2006) for specialist graduates of Fiji origins

While gender appeared to have little impact on decisions to resign, Indo-Fijians were much more likely than Fijians to have resigned from the public sectors (70% versus 31.7%, p = 0.005) or in particular to have migrated out of Fiji (55% versus 17.1%, p = 0.002) (see Fig. 2). One factor that was particularly strongly associated with retention in the public sectors was completion of a master's qualification (see Fig. 3), with 18 of 21 (85.7%) master's graduates still in the public sectors or temporarily overseas, compared with 15 of 42 (35.7%) diploma-only graduates (p = 0.0002).

### Overseas migrants

Seven (of 20) who had left Fiji to live in other countries and had no intention of returning to work in Fiji in the short term or medium term ("overseas migrants") were interviewed, of whom four were Indo-Fijians and three were Fijians. All seven doctors are actively establishing medical careers overseas. Two of the four Indo-Fijian doctors described leaving primarily because of their spouses' career aspirations or family commitments. The other five doctors, including all three Fijians, described being heavily influenced by the 2000 coup, with concerns expressed about raising their children in a politically unstable environment.

I wanted a good future for them and I can't find it back home, given the political situation that has happened. So, I just thought to myself, 'I don't want my kids to go through this'.

The Indo-Fijian doctors who left after the 2000 coup started arranging migration almost immediately, while the Fijians waited longer to migrate, describing a time of hoping that things would get better. No Indo-Fijian described finances as influencing migration decisions. All of the Fijians, however, cited money as a contributing factor. None of the interviewed doctors described migrating for improved training opportunities.

I'd be lying if I said (money had no influence on our decision), but I think in a small way it did. I don't think our decision was based mostly on that. I think my daughter had a lot to do with our decision to move. But, yeah, I guess when you come and work here and you see the amount of money you earn then you think 'oh well, I think I made the right decision to move' (Fijian migrant).

Five out of seven migrants spoke of having overall enjoyed their work in Fiji. For all three Fijians, however, problems with career structure and difficult working conditions contributed heavily to migration decisions.

We were just sort of squashed with work. I said 'I'd better get out of this place, otherwise there's going to be a lot of pressure. It's not good to our health'. No policies in place in the Ministry for furthering a career. I think the biggest factor was just my frustrations with the Ministry. Yes, I mean, they are not treating locally trained people fairly, that's what I thought (Fijian migrant).

### Private practitioners in Fiji

Four doctors in private practice in Fiji were interviewed. They reported resigning from the public service predominantly because of working conditions' interfering with family life, frustrations over career progression or feeling unable to make positive changes in the public sectors. All took time to consider their options before resigning. Three out of four had considered migrating to Australia or New Zealand, and described how the option of local private practice had contributed to their decisions to remain in Fiji.

I was getting fed up with the administration. I'm no longer needed, and so that's when I decided to move out. It took me a year to make up my mind. What would have kept me there? Well, if they had listened to what I suggested to them, then maybe yes I would have stayed on.

At that time I had never thought of private practice. My main decision was to take time off for my family and from there I would decide what to do, but you know people in Fiji, they know what's happening really, so I got offers from everywhere.

Otherwise I would have been out of here. I would have gone abroad. There's really so much that pushes you away. I was already applying into Brisbane.

While private practice was much more lucrative than public-sector work, this was not mentioned as the main motivation for any of the doctors. Doctors appreciated being able to control their hours, spending more time with their patients, having clinical autonomy and logistical support, with the main trade-offs being the loss of opportunities for further specialist training, and missing the "rich" and varied work in the public hospital.

The work here ... you can't compare it with CWM Hospital, but I like it because I can see my patients with my own time.

I'm making about five times more than what I was making in the hospital. But I think every doctor would like to further develop himself. If there was the opportunity, I probably would return, but given the shambles? From what you hear, I am fearful to return.

### Public sector doctors

Twenty-five doctors in the public sector (21) or temporarily training overseas (4) were interviewed about their decisions to continue in public hospital work. Nineteen of these doctors spontaneously described a "service ethic", relating overall satisfaction with public hospital work and a sense of being needed, in spite of considerable frustrations. Eight doctors spontaneously volunteered that their medical practice was powerfully motivated by a belief in God.

I had intentions of leaving, but I guess my philosophy of medicine is really based on the care that I can give to people and it is not based on finance. I think that's what motivates me every day. It may not be in the best conditions, but you know it's the type of care that you give and in the way that you give it that will make the difference.

There's so many specialists (in Australia), I would be just another specialist among so many, whereas, if I came back I would have skills that I could offer.

So it's based on those Christian principles that I have been able to make a lot of my decisions and also walk through some of the difficult times, and under stress and duress, it has really been God giving me my strength and refuge.

Twenty-one out of 25 public sector doctors described their cultural commitments as being very important. Many described a feeling that "Fiji is home", or an appreciation for the laid-back lifestyle and friendliness in Fiji, or feeling committed to Fiji. Fijians in particular described extended family commitments and taking part in cultural events, and the attraction of exposing their children to Fijian culture. A few public sector doctors described "culture" as the major factor keeping them in Fiji (along with three out of four doctors in private practice).

While cultural commitments can help to keep doctors in Fiji, they do not guarantee retention in the public sectors. For some doctors, a desire to serve their own people was a prominent aspect of their cultural attachments, as described above. Others, while seeing overseas careers as being potentially more satisfying or rewarding, did not want to leave Fiji and were either not attracted to the idea of local full-time private practice or they were waiting to see how their public sector careers would unfold.

The main reason (for staying) is security. You feel foreign if you are the only one in the family there, in the midst of millions or thousands of people who don't know you. You can't go to ask for local help, to socialize, like you feel that 'ok we go to auntie this one, to uncle this one, to grandparents here'. It's the lifestyle, the way people live and work and do things there that is probably not the kind of life that I want to live.

Many public sector doctors mentioned work-related frustrations. Fifteen doctors (60%) described unhappiness and pessimism about their own career progression. Ten doctors (40%) had seriously considered resigning, with about half actively seeking out other employment. They reported disillusionment over the 2000 coup (2), overall frustrations over career progression (1), or insensitive interpersonal treatment by administration or supervisors (7), such as extremely insensitive handling of the promotions process (4), being treated disrespectfully (2), insensitivity to serious financial difficulties (2), and believing that leave had been unreasonably denied (3).

When that happened at the beginning of this year, I accepted for the first time I really seriously considered going. I thought, 'Oh, they don't appreciate me! I'm someone that wants to stay here and this is how they treat me!' So it was a very difficult time for me. I thought, 'Okay, I'll stay on, I'll give it a year and if things didn't work out, perhaps that would be where I'd be looking at going.' You question that maybe that wasn't the right decision to stay on and work here, but I love this place. I love the work, I love the people and I love the atmosphere here.

Overall, postgraduate training at FSMed has succeeded in adding 15 master's-qualified specialists to the public sector workforce, with three more master's graduates stating that they plan to return to Fiji after additional overseas training. This compares to only five Fiji doctors with overseas specialist qualifications currently working in the public sector. Disappointingly, 63.6% of enrolees left training without completing a master's, and their public sector retention (14 of 42) has been low (see Fig. 3).

## Discussion

This study had a number of strengths as well as important limitations. Interviews were carried out with over 50% of doctors who had undertaken specialist training at FSMed, with reasonable representation according to ethnicity, gender, specialty and career stage, and included migrants, doctors in private practice or in the public sectors, as well as doctors who had returned from overseas.

The underrepresentation, however, of those who migrated out of the country (35%) or who left training with a diploma as their highest qualification (33.3%), as well the exclusion of diploma "dropouts" from the study, is an important limitation. The longitudinal involvement of the interviewer for almost a decade in Fiji, as well as her role in helping to establish these courses, is likely to have allowed for a deeper understanding of the situations of the interview participants, though this familiarity could have potentially led to bias and avoidance of some topics by interview participants. The overall narrowness of the study is another limitation, and the experiences of medical students, new medical graduates and doctors in rural and regional areas were not explored. Generalization to other countries may be limited, in particular to more impoverished nations.

This study fits well with previous studies in Fiji [38-42], which have cited limited career structures, a lack of sufficient opportunities for promotion, lack of training opportunities (pre-1998), poor working conditions, heavy workloads, problems with remuneration [4,38] and the lack of a perceived link between hard work and rewards [38]. Financial factors were more prominent for a group of doctors from Fiji, Samoa and Tonga who migrated [41], while the concerns of Indo-Fijian migrants in Sydney over family safety and welfare rather than finances were similar to the current study [4]. In other mostly African-based studies, financial factors and concerns about access to training tended to be more prominent than for the Fiji doctors, while other factors such as heavy workloads, poor working conditions, unsatisfactory career progression, dissatisfaction with health management and concerns about family welfare were similar [13-35].

## Conclusion

This study identifies factors that contribute to retaining specialist doctors in the public sector, as well as factors that contribute to resignations from the public sector to enter private practice or migrate overseas. Additionally, it provides some support for the view that in-country or regional specialist training can lead to increased retention of a local specialist workforce, with 15 locally-trained specialists (master's graduates) working in the Fiji public sector by 2006, as compared to only five local specialists who had trained overseas.

The overall impact of the availability of postgraduate training on retention of doctors in the public sector is less clear. From 1996 [38] to 2006 [7], local doctor numbers have increased from 202 (57.2% of 353 established posts) to 251 (61.8% of 406 established posts), while expatriate numbers have fallen from 112 (31.7% of 406 posts) to 90 (16.0% of 353 posts). These data are from different sources, however, and may not be completely comparable. Although the situation has improved somewhat, it is unclear how much of the improvement can be attributed to the availability of postgraduate training and how much is related from the expansion of undergraduate class sizes at FSMed during those years.

One important aspect that this study adds to the literature is the description of a complex career decision-making process, with something of a "composite" emerging from mostly one-off interviews of doctors at different career stages. While public sector work could be rewarding, working conditions were difficult and frustrating, and salaries were low, especially compared to readily-available private work and the many opportunities now available overseas.

"Triggers", such as a political coup, stress-related health problems or episodes of insensitive interpersonal treatment, problems with the promotions process, or even a gradual build-up of frustration over time or increasing stress at home related to work pressures, could lead to a time of "weighing up" whether or not to stay. Diploma-only graduates in particular described weighing up the demands of young family life alongside the difficulties of completing training, and unreliable career progression seemingly unrelated to completing postgraduate training, compared to master's graduates who generally had more frustrations over career progression and not feeling valued.

While some doctors decided quickly to leave, others described wanting to stay in the public sector, and they waited, often longer than a year, hoping things would get better. During this period, promotions were granted or denied, conditions got better or worse or were unchanged, and upsetting interpersonal incidents were rectified or not addressed. Doctors usually stayed where things improved, and if things didn't improve, some left, while others, especially master's graduates, decided, after a period of reflection, to stay anyhow. The recognition of a "weighing up" period suggests a "window" during which some who resigned might have been retained if they believed that problems in the health system were being actively addressed.

Even though it could be argued that the same sense of "determination" that allowed doctors to complete a master's could be helping them to remain in the public sector in spite of frustrations, the promising retention of master's graduates nevertheless suggests that interventions to support trainees through to master's graduation may improve overall retention. Possible strategies include rationalizing the academic workload and trying to adequately staff clinical specialty departments so that the workload for trainees is bearable. Ensuring timely and transparent career progression with rewards for completing specialist training may encourage persistence to master's graduation.

Further research is needed, however, to determine whether interventions to address the factors that led to satisfaction and dissatisfaction for these doctors would lead to a positive impact on doctor retention in public system. Overall, this study provides some support for the view that local or regional postgraduate training may increase retention of doctors. Attention to career pathways and other sources of frustration, in addition to encouragement to complete training, should increase the likelihood of such programmes reaching their full potential.

## Abbreviations

FSMed: Fiji School of Medicine; Mmed: Master of Medicine (specialist qualification); CWM: Colonial War Memorial Hospital (in Suva, Fiji).

## Competing interests

I, Kimberly Oman (principal author) have the following conflicts of interest: I worked at Fiji School of Medicine from 1998 to 2001 and was employed initially by the Fiji School of Medicine and later by AusAID through the Royal Australasian College of Surgeons, which was contracted to establish postgraduate training in Fiji. Part of this study was funded by consultancy fees from the Royal Australasian College of Surgeons in 2002 for two follow-up visits to oversee the progress of the postgraduate training in internal medicine. Neither the Fiji School of Medicine as an institution (apart from individuals as co-authors or supportive colleagues) nor AusAID had input into the planning, data collection, analysis and interpretation of data, in the writing of the report, or in the decision to submit the paper for publication. I have no other conflicts of interest to declare.

I, Robert Moulds (submitting author), have the following conflicts of interest: Before being appointed Professor of Medicine at the Fiji School of Medicine, I was the external advisor for the establishment of the internal medicine component of the AusAID-funded postgraduate programme at the FSMed. I have no other conflicts of interest to declare.

I, Kim Usher, have no conflicts of interest to declare.

## Authors' contributions

KO had full access to all the data in the study, planned the study, carried out the interviews, carried out data analysis and drafted this paper for circulation to other co-authors. RM suggested the study topic and provided guidance and support in the planning and carrying out the fieldwork in Fiji, assisted with analysis of quantitative data, and provided editorial comment on the drafts. KU, as PhD supervisor, had major input into the design of the study and provided oversight of the qualitative data analysis and drafts of the study findings. All authors read and approved the final manuscript.
